# Supplementary material for: Nationwide implementation of the non-invasive prenatal test: Evaluation of a blended learning program for counselors
Source: PLoS One. 2022 May 2;17(5):e0267865. doi: 10.1371/journal.pone.0267865 (PMC9060360; doi:10.1371/journal.pone.0267865)
Supplement: S1 Appendix — (DOCX) [file pone.0267865.s001.docx]

### **Appendix 1 Dutch Setting: Prenatal anomaly screening and TRIDENT studies**

| In the Netherlands, prenatal aneuploidy and structural anomaly screening requires a governmental license, according to the Dutch Population Screening Act, aiming to protect participants from potentially harmful screening [22]. In 2014, a license was granted to the Dutch NIPT Consortium to offer NIPT to women with an increased risk for the common trisomies based on the first-trimester combined test (FCT) (risk >1:200) or medical history as part of the TRIDENT-1 study (Trial by Dutch laboratories for Evaluation of Non-Invasive Prenatal Testing) [19]. In April 2017, a license was given to add NIPT as a first-tier aneuploidy screening test to the Dutch prenatal screening program (TRIDENT-2 study) [23]. Since then, all eligible women can elect either NIPT or FCT as a first-tier screening test at comparable costs (€175 for NIPT and €168 for FCT in 2017) [24]. Women who choose NIPT further select between receiving results only for chromosomes 21, 18, and 13, or receiving results that include the other autosomes (additional findings where abnormalities of all chromosomes are tested). Sex chromosomes are not analysed [18]. Counselors (besides sonographers and gynecologists, mostly midwives (85%)) have to be under contract by one of the eight Regional Centers for prenatal screening (RCs) to offer counseling for prenatal anomaly screening [3,25]. These certified counselors help pregnant women and their partners non-directive decide whether or not to opt for first-trimester aneuploidy screening, and if so, which test to choose: FCT or NIPT. Women can also choose to have the Fetal structural Anomaly Scan (FAS) around 20 weeks of gestational age. To stay up to date, counselors must participate in continuing professional education as well as in tailored, up-to-date education about, for example, developments regarding counseling for prenatal anomaly screening and on relevant aspects of NIPT [6]. Since 1 September 2021, a first trimester fetal anomaly scan (FTFAS) was added to the Dutch screening program as part of the IMplementation of fIrst Trimester Anomaly Scan study (IMITAS study). Since 1 October 2021 the FCT was removed from the Dutch prenatal screening program, because of low uptake-numbers. |
| --- |
