# Supplementary material for: Nationwide implementation of the non-invasive prenatal test: Evaluation of a blended learning program for counselors
Source: PLoS One. 2022 May 2;17(5):e0267865. doi: 10.1371/journal.pone.0267865 (PMC9060360; doi:10.1371/journal.pone.0267865)
Supplement: S2 Appendix — (DOCX) [file pone.0267865.s002.docx]

### **Appendix 2 Development of the resources for the blended learning program**

The development of the blended learning program for the introduction of NIPT as first-tier screening test for aneuploidies (e.g. Down-, Edwards- and Patau syndrome), which was implemented as part of an existing educational program for counselors, began by exploring counselors' knowledge gaps existing after the first educational activities about NIPT during the TRIDENT-1 study in April 2014, when NIPT was available as a second-tier test (see context Appendix 1) [6], and by utilising the results of a study about the role of genetics in primary care [26]. The top three knowledge gaps that these studies revealed were: test characteristics of NIPT, inclusion and exclusion criteria for NIPT, and characteristics of the anomalies detected by the test. Based on these findings, the Centre for Population Screening of the National Institute for Public Health and the Environment (RIVM-CvB) and the Regional Centers for prenatal screening (RCs), in cooperation with the NIPT consortium, developed and organised an educational program for Dutch counselors. The intention was to make a flexible and effective framework using multiple didactical methods, educational media, and settings (blended learning) to achieve the first three levels of Miller's Pyramid [21]. The resulting blended learning program was implemented by the RCs, alongside the introduction of NIPT as the first-tier test in April 2017, and included: 1) an e-learning consisting of ten modules including a knowledge examination that needed to be passed (with 3 attempts possible); 2) a lesson plan of a 4-hour face-to-face seminar and accompanying PowerPoint presentations about the test characteristics of NIPT, NIPT within the Dutch prenatal screening program, conditions screened for (Down, Edwards and Patau syndrome), and organizational aspects of the NIPT and the counseling; 3) guidelines for counseling consisting of medical test information in text and Option Grid decision guides, as well as suggestions for communication with clients; 4) factsheets to support clients’ understanding during counseling and websites with helpdesks for both counselors and clients [27], and 5) a skills training including role-playing (Fig1). All educational resources and additional client information (both web-based and written leaflets and online decision aid for clients) were developed by a panel of experts assembled by the RIVM-CvB and included a representative of a patient organisation, midwife counselors, gynecologists, clinical geneticists, educators, communication experts, and researchers in the field of counseling for prenatal anomaly screening. Based on the assessment of counselors’ knowledge measured during T0, the PowerPoints presentations could be tailored to specific counselors' needs during the seminars the RCs provided.
