# Supplementary material for: Nationwide implementation of the non-invasive prenatal test: Evaluation of a blended learning program for counselors
Source: PLoS One. 2022 May 2;17(5):e0267865. doi: 10.1371/journal.pone.0267865 (PMC9060360; doi:10.1371/journal.pone.0267865)
Supplement: S3 Appendix — (PDF) [file pone.0267865.s003.pdf]

The Dutch NIPT Consortium

The Dutch NIPT Consortium consists of obstetric care givers, laboratory specialists and other professionals from:

Amsterdam UMC*

Clinical Genetics:

Dr. E.A. Sistermans (also for the Dutch Association of Clinical Genetic Diagnostic Laboratories (VKGL))
Prof. L. Henneman
Dr. A. Polstra
E. Voorhoeve MSc
S.L. Zelderen-Bhola MSc
Dr. E.M.J. Boon
Dr. M.P.R. Lombardi
I.M.C. Bakker MSc
E.J. Bradley BSc
C. Louwerens-Zintel BSc
M. Smit BSc
Dr. M.C. van Maarle
M.B. Tan-Sindhunata MSc
K. van der Meij MSc
Prof. H. Meij

Obstetrics and Gynecology:

Dr. C.J. Bax (also for the Dutch Organization of Obstetrics and Gynecology (NVOG))
Prof. E. Pajkrt
Dr. I.H. Linskens

Midwifery Science AVAG:

Dr. L. Martin
Dr. J.T. Gitsels-van der Wal

Erasmus Medical Center, Rotterdam*

Clinical Genetics:

Dr. R.J.H. Galjaard (also for the Dutch Association of Clinical Geneticists (VKGN))
Dr. D. Van Opstal
Dr. M.I. Srebniak
Dr. F.M. Sarquis Jehee
I.H.I.M Hollink MSc
Dr. F. Sleutels
W. de Valk BSc
W.H. Deelen BSc
Dr. A.M.S. Joosten
Dr. K.E.M. Diderich
M.E. Redeker

Obstetrics and Gynecology:

Dr. A.T.J.I Go
Dr. M.F.C.M. Knapen
Dr. S. Galjaard
Dr. A.K.E. Prinsen

Information & Technology:

A.P.G. Braat

Leiden University Medical Center

Clinical Genetics:

Dr. M.J.V. Hoffer
Dr. N.S. den Hollander

Obstetrics and Gynecology:

Dr. E.J.T. Verweij
Dr. M.C. Haak

Maastricht University Medical Center*

Clinical Genetics:

Dr. M.V.E. Macville
Dr. S.J.C. Stevens
Dr. A. van der Wijngaard
Dr. H. Scheffer
L.H. Houben
M.A.A. van Esch-Lennarts BSc
Prof. C.E.M. de Die-Smulders
Prof. H. Brunner

Obstetrics and Gynecology:

M.J. Pieters MD (also for the Regional Centers Prenatal Screening in The Netherlands)
Dr. A.B.C. Coumans

Radboud University Medical Center, Nijmegen

Clinical Genetics:

Dr. D.F.C.M. Smeets
Dr. B.H.W. Faas
Dr. D.E.M. Oldeweghuis
T. Hofste BSc
I. Derks-Prinsen BSc
Dr. I. Feenstra

Obstetrics and Gynecology:

Dr. E. Sikkel

University Medical Center Groningen

Clinical Genetics:

Dr. R.F. Suijkerbuijk
Dr. B. Sikkema-Raddatz
Prof. I.M. van Langen
Dr. K. Bouman

Obstetrics and Gynecology:

L.K. Duin MD

University Medical Center Utrecht

Clinical Genetics:

Dr. G.H. Schuring-Blom
Dr. K.D. Lichtenbelt

Obstetrics and Gynecology:

Prof. M.N. Bekker

The Royal Dutch Organization of Midwives (KNOV)

Dr. A.J.E.M. van der Ven

VSOP Dutch Genetic Alliance

E. van Vliet-Lachotzki MD

Erfocentrum, Dutch National Genetic Resource and Information Center

J. Pot

Dutch NIPT Consortium, project manager

S. van 't Padje

NIPT laboratories

Date version: March 2021
